# Endoplasmic reticulum stress in *amelogenesis imperfecta* and phenotypic rescue using 4-phenylbutyrate

**DOI:** 10.1093/hmg/ddt642

**Published:** 2013-12-20

**Authors:** Steven J. Brookes, Martin J. Barron, Ray Boot-Handford, Jennifer Kirkham, Michael J. Dixon

**Affiliations:** 1 Department of Oral Biology, LeedsDental Institute, University of Leeds, Clarendon Way, Leeds LS2 9LU, UK; 2 Faculty of Life Sciences and; 3 School of Dentistry, Manchester Academic Health Sciences Centre, University of Manchester, Michael Smith Building, Oxford Road, Manchester M13 9PT, UK

## Abstract

Inherited diseases caused by genetic mutations can arise due to loss of protein function. Alternatively, mutated proteins may mis-fold, impairing endoplasmic reticulum (ER) trafficking, causing ER stress and triggering the unfolded protein response (UPR). The UPR attempts to restore proteostasis but if unsuccessful drives affected cells towards apoptosis. Previously, we reported that in mice, the p.Tyr64His mutation in the enamel extracellular matrix (EEM) protein amelogenin disrupts the secretory pathway in the enamel-forming ameloblasts, resulting in eruption of malformed tooth enamel that phenocopies human *amelogenesis imperfecta* (AI). Defective amelogenin post-secretory self-assembly and processing within the developing EEM has been suggested to underlie the pathogenesis of X chromosome-linked AI. Here, we challenge this concept by showing that AI pathogenesis associated with the p.Tyr64His amelogenin mutation involves ameloblast apoptosis induced by ER stress. Furthermore, we show that 4-phenylbutyrate can rescue the enamel phenotype in affected female mice by promoting cell survival over apoptosis such that they are able to complete enamel formation despite the presence of the mutation, offering a potential therapeutic option for patients with this form of AI and emphasizing the importance of ER stress in the pathogenesis of this inherited conformational disease.

## INTRODUCTION

Membrane proteins and proteins destined for secretion are translated and simultaneously translocated into the rough endoplasmic reticulum (ER) by ER-bound ribosomes for trafficking to target destinations. During translocation and subsequent trafficking, nascent proteins begin to fold, attaining specific conformations that determine protein functionality (1). To aid this process, ER-resident chaperones bind client proteins and promote correct folding by minimizing mis-folding and aggregation in a biologically relevant timescale (2). However, physical or chemical stressors, genetic mutations and advanced age are associated with an increased incidence of protein mis-folding. Several major human diseases have been attributed to protein mis-folding. These ‘conformational diseases’, which include Alzheimer's disease, Huntington's disease, Parkinson's disease, atherosclerosis, cystic fibrosis and type 2 diabetes, arise when proteins fail to achieve their correct conformation and aggregate in the ER due to exposure of hydrophobic domains (3,4). Unfolded or mis-folded proteins are detected and destroyed through ER-associated degradation (5) or autophagy (6), and thus ER homeostasis is maintained. However, if the capacity of the folding and degradation machinery is exceeded, mis-folded proteins accumulate in the ER and cause ER stress (7). Quality control systems in the ER centred on the transmembrane receptors/transducers IRE1, PERK and ATF6, constantly monitor the secretory cargo and on detection of an imbalance of mis-folded proteins trigger three intracellular pathways that together comprise the unfolded protein response (UPR). The UPR attempts to alleviate ER stress by (i) decreasing protein synthesis, (ii) increasing the size of the ER, (iii) increasing the synthesis of ER chaperones and (iv) up-regulating components of the ER-associated degradation pathway. If homeostasis cannot be restored and ER stress is prolonged, the UPR can direct cells towards apoptosis (7,8).

Recently, we described a p.Tyr64His mutation in the developing enamel extracellular matrix (ECM) protein amelogenin in mice that resulted in eruption of malformed tooth enamel that exhibited severely compromised mechanical properties thereby mirroring human *amelogenesis imperfecta* (AI) (9). AI is a common genetic disorder with an incidence as high as 1 in 700 live births (10) which results in considerable morbidity, pain and low self-esteem due to poor aesthetics (Supplementary Material, Fig. S1) (11). Using this mouse model, we demonstrated that the p.Tyr64His mutation apparently disrupts the secretory pathway of the enamel-forming ameloblasts and speculated that this phenomenon might lead to ER stress which has been shown to be a factor in other inherited skeletal connective tissue diseases involving mutations in secreted ECM proteins (12).

In the present study, we tested the hypothesis that the p.Tyr64His amelogenin mutation results in an inherited conformational disease that manifests as AI in this mouse model. Our data indicate that in the presence of the mutation, amelogenin accumulates intracellularly, inducing ER stress-related apoptosis in the ameloblasts. As such, this is the first report classifying AI as a conformational disease. Importantly, we also demonstrate that 4-phenylbutyrate dramatically rescues the phenotype in female mice that are heterozygous for the mutation. Treatment options for AI are limited and rely on costly and invasive restorative procedures. 4-Phenylbutyrate is already licensed for the treatment of inborn errors of the urea cycle (unrelated to ER stress) where it provides an alternative excretory route for the renal elimination of amino acids (13). We suggest that 4-phenylbutyrate may be effective in the treatment of certain types of AI and other skeletal connective tissue diseases related to ER stress associated with the intracellular trafficking of mutated ECM proteins.

## RESULTS

### The ER secretory pathway is impaired in p.Tyr64His amelogenin mutants

**Figure 1.**
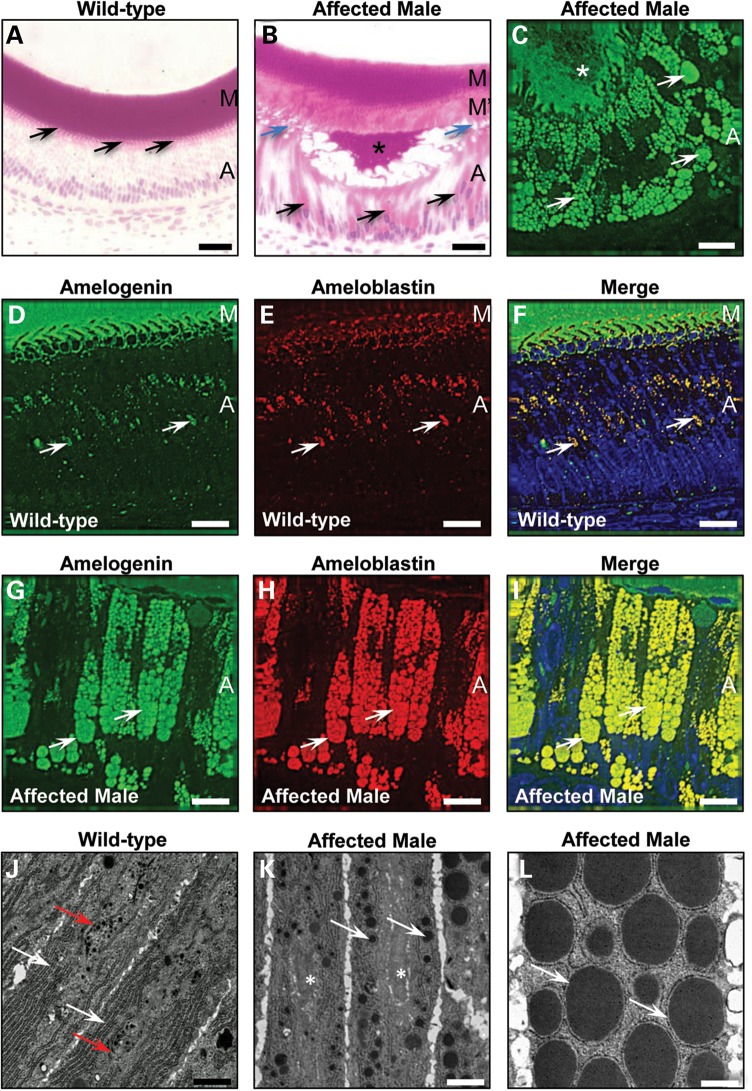
Secretory stage ameloblasts of affected male mice manifest a defect in the amelogenin secretory pathway. (**A**) Wild-type secretory stage ameloblasts (*n* = 3) are organized as a tall columnar epithelium with secretory Tomes' processes that inter-digitate with the enamel ECM (arrows). (**B**) In contrast, affected male secretory ameloblasts (*n* = 3) are shorter, lose their Tomes' processes (blue arrows), exhibit highly eosinophilic cytoplasm (black arrows) and secrete less enamel ECM. The secreted ECM shows a biphasic eosin staining pattern with the secreted matrix (M′) deposited later in the secretory stage being less eosinophilic than that produced at the earliest stages of amelogenesis (M). (**C**) The cytoplasmic vesicles observed in the secretory ameloblasts of affected male mice (*n* = 3) are strongly immuno-reactive for amelogenin (arrows) as is the secreted enamel ECM (asterisk). Scale bars: (A) and (B) 20 μm; (C) 10 μm. (**D–F**) Dual immunofluorescence for amelogenin and ameloblastin demonstrates that both proteins co-localize in the cytoplasm (arrows) of wild-type mice (*n* = 3). Nuclei are stained with DAPI (blue). A, ameloblasts; M, matrix. Scale bars: 10 μm. (**G–I**) Dual immunofluorescence for amelogenin and ameloblastin demonstrates that both proteins co-localize in the large cytoplasmic vesicles (arrows) (*n* = 3). Nuclei are stained with DAPI (blue). A, ameloblasts. Scale bars: 10 μm. (**J–L**) Transmission electron microscopy of secretory stage ameloblasts. (J) In wild-type mice (*n* = 3), the ER is organized longitudinally and arranged peripherally close to the plasma membrane (white arrows), while the Golgi apparatus and secretory granules are located towards the centre of the cell (red arrows). (K) In early secretory ameloblasts of affected male mice (*n* = 3), protein-containing inclusions form towards the periphery of the cell in the same region as the ER (white arrows) while the central area of the cell lacks a recognizable Golgi apparatus but contains dilated cisternae (asterisk). (L) The membrane-bound inclusions in affected male mouse ameloblasts enlarge progressively until they occupy the majority of the cellular volume (arrows). Scale bars: (D) and (E) 1 μm; (F) 500 nm.

**Figure 2.**
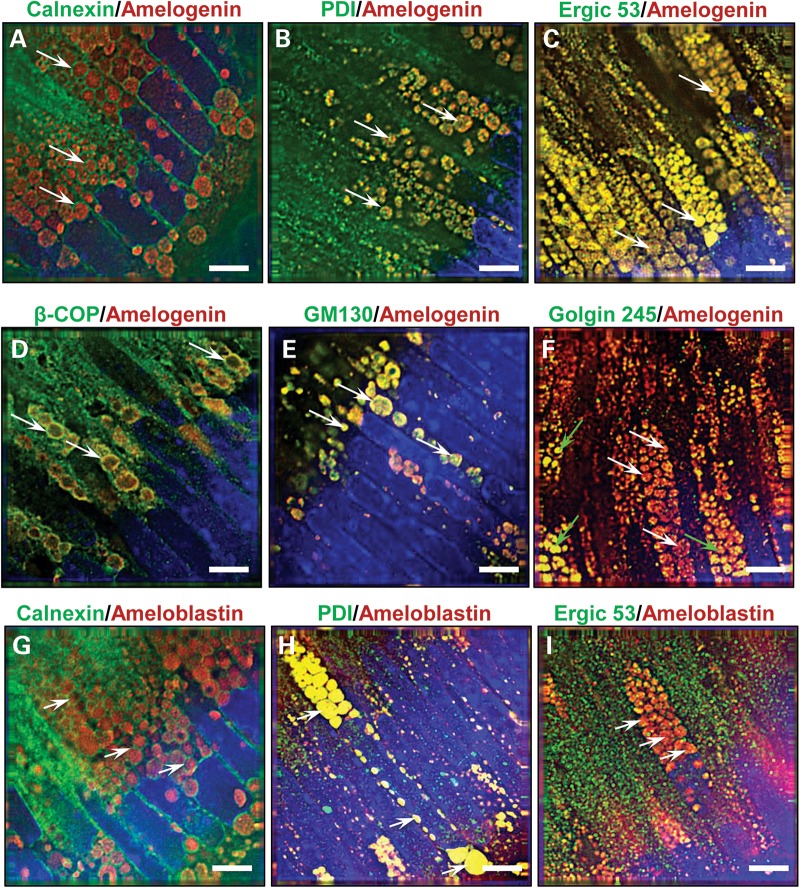
Characterization of the cytoplasmic vesicles observed in affected male mice. (**A–F**) Dual immuno-staining for amelogenin (red fluorescence) or (**G–I**) ameloblastin (red fluorescence) and components of the secretory pathway (green fluorescence) in affected male mice (*n* = 4) secretory ameloblasts indicate arrest of amelogenin trafficking at a stage prior to the *trans-*Golgi. (A) The ER membrane protein calnexin circumscribes the amelogenin-containing vesicles (arrows), while the ER luminal molecule protein disulphide isomerase (B) co-localizes with amelogenin within the vesicles (arrows). (C) Immuno-reactivity for the ER/Golgi intermediate compartment molecule Ergic 53 also co-localizes with amelogenin in the vesicles (arrows), whereas (D) β-COP, a component of COP1 vesicles found at the *cis-*Golgi and ER/Golgi intermediate compartment, co-localizes with amelogenin at the periphery of the vesicles (arrows). (E) The *cis-*Golgi protein GM130 co-localizes with amelogenin within the vesicles (arrows). (F) Golgin 245, a *trans-*Golgi protein, showed a variable pattern of immuno-reactivity; in most instances, little amelogenin co-localization was observed in the vesicles (white arrows), although, less typically, partial or complete co-localization was seen (green arrows). (G) Dual immunofluorescence for ameloblastin and the ER membrane protein calnexin indicates that calnexin circumscribes the inclusions (arrows). (H) Similar analyses using antibodies directed against ameloblastin and the ER luminal molecule protein disulphide isomerase (PDI) indicate co-localization within the vesicles (arrows). (I) In contrast, dual immunofluorescence with ameloblastin and Ergic 53, a protein found in the ER/Golgi intermediate compartment, demonstrates distinct localization within the ameloblasts (arrows). Nuclei are stained with DAPI (blue). Scale bars: 5 μm.

Histological analysis demonstrated that the cytoplasm in secretory ameloblasts of affected mice was abnormally engorged with multiple eosinophilic vesicles that were immuno-positive for amelogenin and ameloblastin; the second most abundant enamel ECM protein (Fig. 1A–I). Ultrastructural analysis suggested that these vesicles formed within the ER and were associated with a reduction in the volume of the Golgi apparatus (Fig. 1J–L). Immunofluorescence analysis using antibodies to ER resident proteins, proteins of the ER/Golgi intermediate compartment/*cis*-Golgi and *trans*-Golgi showed that all but the *trans*-Golgi proteins localized to the intracellular vesicles of affected ameloblasts suggesting a collapse of the normal secretory pathway (Fig. 2A–I; Supplementary Material, Fig. S2A–I).

### The p.Tyr64His amelogenin mutation induces an ER stress response in ameloblasts

**Figure 3.**
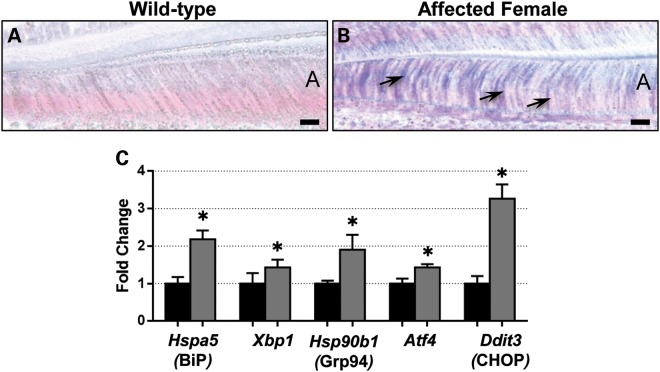
The p.Tyr64His mutation in amelogenin results in ER stress increased intermolecular interactions of mutant amelogenins. (**A** and **B**) *In situ* hybridization indicates that the secretory stage ameloblasts of affected female mice (*n* = 3) (B) express *Hspa5* (BiP) more strongly than wild-type littermates (*n* = 3) (A). Scale bar: 10 μm. (**C**) Real-time PCR analysis of micro-dissected secretory enamel organs indicates that the levels of ER stress response genes are elevated in affected mice (*n* = 4) compared with wild-type (*n* = 4) littermates. *P* = 0.014, *Hspa5*, *Hspa90b1*, *Atf4* and *Ddit3*; *P* = 0.043 *Xbp1*.

To determine whether affected ameloblasts were under ER stress, we performed *in situ* hybridization and quantitative RT–PCR to investigate expression of ER stress genes. *In situ* hybridization indicated increased *Hspa5* expression, which encodes BiP, a key component of the ER stress response (14) in secretory ameloblasts of affected mice compared with their wild-type littermates (Fig. 3A and B). Similarly, quantitative RT–PCR analysis of enamel organs containing secretory ameloblasts showed elevated expression levels of ER stress genes *Hspa5*, *Xbp1*, *Hspa90b1* (Grp94), *Atf4* and *Ddit3* (Chop) in affected mice compared with their wild-type littermates (Fig. 3C).

### 4-Phenylbutyrate alleviates ER stress-induced apoptosis in p.Tyr64His transfected COS-7 cells

To determine whether 4-phenylbutyrate was able to alleviate the ER stress associated with the p.Tyr64His mutation, we co-transfected COS-7 cells with p.Tyr64His amelogenin and wild-type ameloblastin which resulted in a significant increase in apoptosis (*P* < 0.0001) compared with controls transfected with wild-type amelogenin or wild-type ameloblastin alone or in combination. Crucially, apoptosis was abrogated by addition of 0.5 mm 4-phenylbutyrate (Supplementary Material, Fig. S3A). In parallel experiments, the p.Pro70Thr amelogenin mutation which underlies AI in a human kindred (15), generated a similar increase in apoptosis that was also abrogated by 4-phenylbutyrate (Supplementary Material, Fig. S3B).

### 4-Phenylbutyrate improves the gross appearance of incisor teeth in female mice carrying the p.Tyr64His amelogenin mutation

**Figure 4.**
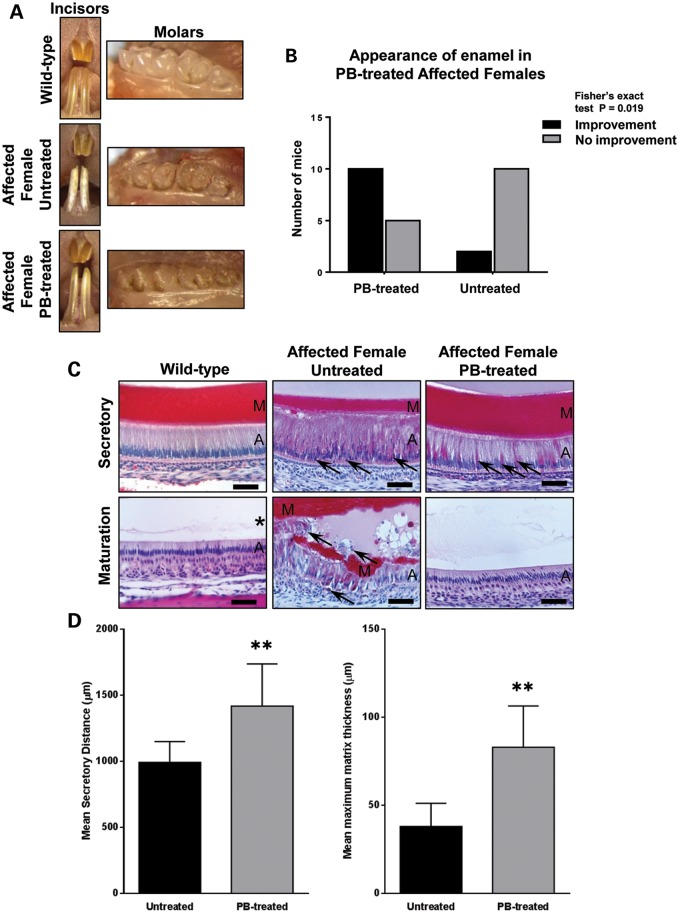
Phenotypic rescue using 4-phenylbutyrate *in vivo*. (**A**) The incisor teeth of wild-type mice are smooth and opalescent while those of affected female mice exhibit a chalky-white, opaque appearance with roughened/pitted surfaces. 4-Phenylbutyrate treatment restores the appearance of affected female mice incisors to wild type. In contrast, phenotypic rescue of the molar teeth, which do not erupt continuously, is not observed. (**B**) Qualitative assessment of the enamel appearance by independent researchers. This difference is statistically significant (Fisher's exact test; *P* = 0.019). (**C**) Histological analysis indicates that the tall columnar secretory stage ameloblasts of wild-type mice secrete a thick, eosinophilic, enamel ECM while the maturation stage ameloblasts are shorter and the enamel ECM has been digested (asterisk). The secretory ameloblasts of untreated affected female mice are disorganized with intensely eosinophilic cytoplasm and numerous apoptotic cells (arrows). A thinner enamel matrix has been secreted. At the maturation stage, multicellular masses of ameloblasts have formed around retained enamel ECM. Many of the ameloblasts are apoptotic (arrows). By contrast, the histological appearance of the enamel organ of 4-phenylbutyrate-treated affected female mice closely resembles that of wild-type mice with only occasional secretory ameloblasts displaying a strongly eosinophilic cytoplasm (arrows). A, ameloblasts; M, enamel matrix. Scale bars: 50 μm. (**D**) Morphometric analysis of the enamel organ indicates that the length of the ameloblast secretory zone (*P* = 0.0031) and the maximum thickness of the enamel ECM (*P* = 0.0064) deposited is increased in affected female mice treated with 4-phenylbutyrate. PB-treated mice, *n* = 15; control mice, *n* = 12. PB, 4-phenylbutyrate. Error bars represent the standard deviation of the mean for all datasets.

In light of the *in vitro* transfection studies, we administered a diet containing 7 g/kg sodium-4-phenylbutyrate to wild-type and affected mice for 10 weeks while monitoring their continuously erupting incisor teeth. Wild-type incisors were smooth and opalescent throughout the experiment with no apparent differences between mice maintained on the control diet and those receiving 4-phenylbutyrate (Fig. 4A). At the outset of the experiment, the incisor teeth of affected female mice were fractured/worn and uneven, exhibiting a chalky-white appearance with roughened/pitted surfaces phenocopying human AI (Fig. 4A). During the course of the experiment, the incisors of both groups continued to erupt. The majority of 4-phenylbutyrate-treated affected female mice showed a marked improvement in the appearance of their incisors over the course of the experiment with the poor-quality enamel typically being replaced by translucent, shiny, orange-coloured enamel characteristic of wild-type mice (Fig. 4A and B). In contrast, the incisors of affected male mice treated with 4-phenylbutyrate failed to show a similar improvement presumably because they are hemizygous for amelogenin and therefore produce exclusively mutant protein. The defective molar enamel in affected male and female mice was unaffected by 4-phenylbutyrate treatment (Fig. 4A). This observation was predicted since these teeth do not erupt continuously and would have already completed their developmental program prior to the mice receiving 4-phenylbutyrate.

### 4-Phenylbutyrate improves the p.Tyr64His phenotype at the histological level

**Figure 5.**
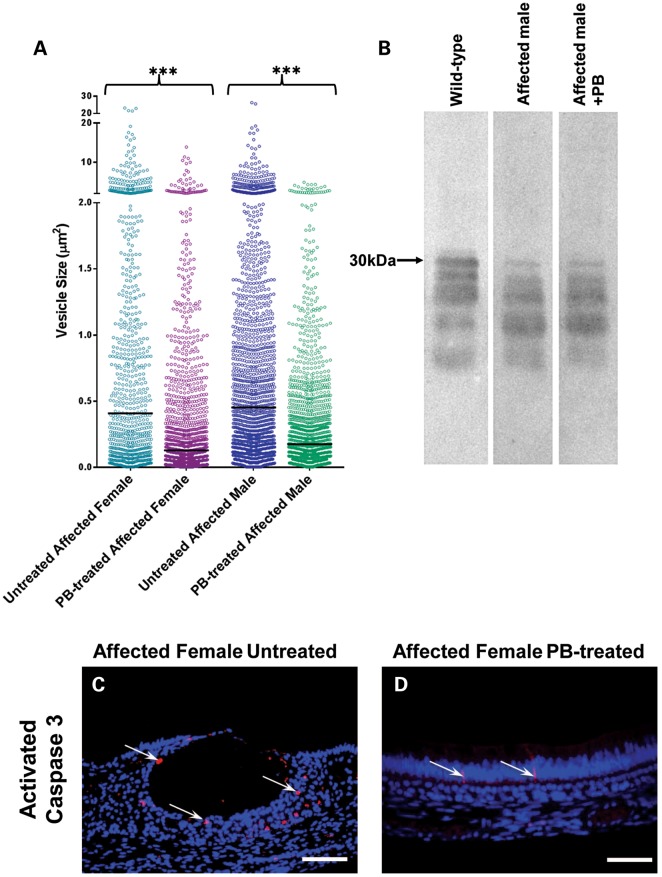
Effect of PB treatment on the secretory stage enamel organ. (**A**) Effect of 4-phenylbutyrate (PB) treatment on the size of cytoplasmic vesicles in mutant mice. Ameloblastin immuno-staining was used to delineate the cytoplasmic vesicles in the secretory stage ameloblasts of untreated and PB-treated affected female and affected male mice with image analysis being used to determine their area. As indicated in the scattergram, the distribution of vesicle area shifted to lower values in PB-treated affected female and affected male mice compared with their untreated littermates. Four mice were examined, and three images analysed in each group. Comparison of the median values using Kruskal–Wallis analysis followed by a Dunn's multiple comparison test showed highly significant differences in vesicle size between untreated and PB-treated mice of both genotypes (*P* < 0.0001). In contrast, there was no statistically significant differences in median vesicle size between PB-treated male and PB-treated female mice (*P* = 0.064). (**B**) Western blotting of secreted matrix proteins suggests that PB does not completely relieve secretory impairment. Two wild-type, four PB-treated affected male and two untreated affected male mice were analysed. (**C** and **D**) Activated caspase 3 immuno-staining was used to identify secretory stage ameloblasts undergoing apoptosis in untreated and PB-treated affected female mice. In untreated mice (C), numerous apoptotic cells were observed (arrows) while in the PB-treated affected female mice (D), fewer apoptotic cells were detected (arrows). Three mice were examined in each group. Scale bars: 100 μm.

Histologically, wild-type mouse incisors showed typically tall secretory-stage ameloblasts associated with an eosinophilic enamel ECM (Fig. 4C). As the ameloblasts entered the maturation stage, during which rapid crystal growth appears and mineral content dramatically increases, they shortened and the proteins were removed from the enamel ECM as evidenced by the loss of staining (Fig. 4C). In contrast, secretory ameloblasts in affected female mice exhibited intracellular eosinophilic staining, indicating retained matrix protein, and the length of the secretory zone and thickness of the associated enamel ECM were reduced compared with wild-type mice (Fig. 4C and D). Maturation stage ameloblasts in affected female mice were highly disorganized with substantial ECM retained in the underlying enamel (Fig. 4C). Similar analyses of affected female mice that received 4-phenylbutyrate indicated that these abnormalities were abolished, with the ameloblasts and ECM being histologically indistinguishable from wild-type mice (Fig. 4C and D). Image analysis indicated that the size of the cytoplasmic vesicles of affected mice was reduced significantly by 4-phenylbutyrate treatment (Fig. 5A).

### 4-Phenylbutyrate appears to rescue the p.Tyr64His phenotype by inhibiting apoptosis

Western analysis of secretory stage ECM from affected male mice revealed that amelogenin secretion was still impaired despite 4-phenylbutyrate treatment, suggesting that the therapeutic value of 4-phenylbutyrate was not linked to a restoration of the normal secretory pathway in affected ameloblasts (Fig. 5B). We could not, however, analyse affected female mice in this way; in these mice, ∼50% of the ameloblasts are expressing wild-type amelogenin, and the enamel matrix is rich in normal amelogenins. Against this amelogenin-rich background, it is impossible to visualize any effect of 4-phenylbutyrate on the secretory activity of the remaining 50% of ameloblasts expressing mutant amelogenin.

To determine whether the observed therapeutic value of 4-phenylbutyrate was linked to modulation of the UPR such that ameloblast survival was promoted over apoptosis, we compared levels of activated caspase 3 in ameloblasts of affected mice with or without 4-phenylbutyrate treatment. In the absence of 4-phenylbutyrate, large numbers of ameloblasts were immuno-positive for activated caspase 3 whereas treatment with 4-phenylbutyrate reduced activated caspase 3 levels to those seen in wild-type mice, indicating that 4-phenylbutyrate was indeed inhibiting ameloblast apoptosis in affected animals (Fig. 5C and D and Supplementary Material, Fig. S4).

### 4-Phenylbutyrate treatment restores enamel micro-architecture and mineral density in affected female mice

**Figure 6.**
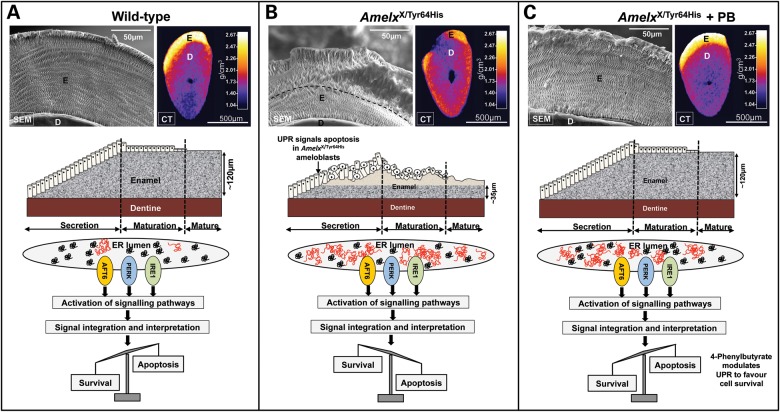
Characterization of phenotypic rescue achieved with 4-phenylbutyrate. (**A**) SEM of wild-type incisors showing ordered decussating enamel rod structure (**E**) and transverse and longitudinal CT scans (calibrated for mineral density) showing the dense enamel layer overlying dentine (**D**). The cartoon shows enamel being deposited by ameloblasts during the secretory stage as the tooth erupts left to right. Maturation stage ameloblasts shorten as the enamel attains its final mineral density. To accommodate the large secretory cargo transiting the ER and spontaneously mis-folded/aggregated wild-type amelogenin (depicted in red), the UPR is activated, IRE1 is activated in secretory ameloblasts (16) and cell survival is promoted. (**B**) The enamel of affected female mouse incisors is biphasic with an inner layer of apparently normal decussating enamel (∼35 µm thick) and an outer layer containing fewer, disorganized prisms. Outer enamel is frequently missing having fractured along the inner–outer enamel boundary (dashed line). The increase in mis-folded/aggregated mutated amelogenin accumulating in the ER is tolerated initially and enamel secretion is unaffected. However, after ∼35 µm of enamel is deposited, the increasing burden of ER stress drives the UPR towards an apoptotic endpoint. Those ameloblasts expressing p.Tyr64His amelogenin die, resulting in disruption of the ameloblast layer which compromises subsequent enamel deposition. (**C**) 4-Phenylbutyrate restores the decussating prism structure and appearance of the enamel layer to wild type. Western blotting (Fig. 5B) and cellular retention of eosinophilic vesicles (Fig. 4C) indicate that 4-phenylbutyrate does not greatly improve amelogenin secretion. We therefore suggest that 4-phenylbutyrate modulates the UPR response in favour of survival meaning that a functional ameloblast layer is maintained and enamel deposition proceeds undisturbed even though amelogenin secretion is presumably reduced owing to cellular retention of mutated protein. Three mice were examined in each group. PB, 4-phenylbutyrate.

To assess the effect of 4-phenylbutyrate treatment on the enamel mineral phase in affected animals, we employed high-resolution X-ray computed tomography (CT) and scanning electron microscopy (SEM). CT demonstrated that the incisor enamel of affected female mice was hypomineralized, with the mature enamel exhibiting a roughened, hypoplastic appearance (Fig. 6A and B, and Supplementary Material, Video S1). Treatment with 4-phenylbutyrate dramatically improved both the quantity and quality of enamel in affected female mice, restoring mineral density to that observed in wild-type mice in the majority of cases with no remaining evidence of hypoplasia (Fig. 6C and Supplementary Material, Video S1). SEM showed that the enamel of affected female mice was biphasic with a layer of inner enamel that appeared normal exhibiting the distinctive decussating arrangement of enamel rods characteristic of rodent incisor enamel micro-architecture. In contrast, the outer enamel layer was structurally abnormal with a loss of decussation and a reduction in the number of enamel rods present (Fig. 6A and B). When affected female mice were treated with 4-phenylbutyrate, the whole enamel architecture was dramatically restored to that observed in wild-type mice with decussating enamel rods traversing the full thickness of the enamel without interruption (Fig. 6A and C).

### The effect of 4-phenylbutyrate treatment on affected male mice is limited

In contrast to affected female mice, there was no similar improvement in the gross appearance of incisor enamel in affected male mice following 4-phenylbutyrate treatment. However, 4-phenylbutyrate treatment resulted in a significant increase in the length of the organized secretory zone and matrix thickness in affected males (Supplementary Material, Fig. S5A) and the number of apoptotic cells was reduced (Supplementary Material, Fig. S5B and C). CT and SEM indicated that affected male incisor enamel remained severely hypoplastic with only a superficial layer of rod-free enamel present on the tooth surfaces. In contrast to affected female mice, 4-phenylbutyrate treatment failed to rescue this situation (Supplementary Material, Fig. S5D–F).

## DISCUSSION

A number of serious human diseases are associated with ER stress induced by the mis-folding of newly synthesized proteins as they are trafficked through the ER (4,5,12,17,18). Even under normal circumstances, up to 30% of proteins fail to fold correctly (19). The UPR, acting as a cellular quality control system, protects cells from the cytotoxic effects of mis-folded proteins by increasing the availability of chaperones and/or directing mis-folded proteins to the ER-associated degradation pathway for destruction to restore homeostasis. However, if the amount of mis-folded protein exceeds the capacity of the UPR to maintain homeostasis, the UPR commits the cell to apoptosis (20). The three ER stress transducers IRE1, ATF6 and PERK are activated by rising levels of mis-folded proteins in the ER triggering the UPR. Although not fully understood, integration of the myriad signals cascading from activation of IRE1, ATF6 and PERK dictates whether cell survival or apoptosis is favoured (21).

Previously, we have demonstrated that the p.Tyr64His mutation in murine amelogenin results in dental anomalies that phenocopy X-linked AI observed in humans (9). Here, we show that this phenotype arises due to impairment of the normal ameloblast secretory pathway leading to ER stress and large-scale ameloblast apoptosis rather than as a result of post-secretory amelogenin dysfunction in the developing enamel ECM. Wild-type secretory ameloblasts constitutively utilize the UPR as they exhibit increased levels of activated IRE1 and its downstream target XBP-1 (16), a transcription factor targeting multiple downstream target genes the majority of which are involved in increasing the folding capacity of the ER or with the degradation of proteins accumulating in the ER (22,23). This process is typical of cells with high secretory activity and a large flux of proteins passing through the ER such as ameloblasts, pancreatic β-cells and plasma cells, as it aids physiological adaptation to the large secretory load (14,24). However, the presence of p.Tyr64His mutant amelogenin in the ameloblast secretory cargo clearly exceeds the capacity of the UPR to maintain ameloblast homeostasis as evidenced by the breakdown of the normal secretory machinery and intracellular accumulation of amelogenin in vesicles apparently composed of the disorganized remnants of the ER-Golgi secretory pathway (Figs 1 and 2 and Supplementary Material, Fig. S2).

Our data show that the p.Tyr64His amelogenin mutation is associated with an up-regulation of several UPR-associated genes including *Hspa5*, *Xbp1*, *Hspa90b1*, *Atf4* and *Ddit3* (Fig. 3) presumably induced by the observed ER stress and indicating that the UPR has been further activated to levels over and above that deployed for cell homeostasis in wild-type ameloblasts. *Ddit3* encodes the transcription factor Chop which is induced by activation of IRE1, PERK and ATF6 that then activates downstream apoptosis pathways (25). Together with the presence of an activated caspase cascade (Fig. 5C and Supplementary Material, Fig. S5C), the data indicate that p.Tyr64His amelogenin aggregates, stalled in the secretory pathway, overwhelm the ability of the UPR to restore homeostasis and instead the UPR drives the ameloblasts towards apoptosis.

Amelogenesis involves the incremental secretion of a protein-rich ECM onto the pre-formed underlying dentine by the ameloblast monolayer (26). The partially mineralized ECM is primarily composed of amelogenin and its extracellularly derived processing products. Once the full thickness of the enamel layer is secreted, delineating final enamel thickness, the ECM proteins are degraded. The enamel then enters a maturation stage as the ameloblasts pump mineral ions into the ECM permitting the thin, pre-existing crystals to grow in width and thickness so that the tissue is eventually ∼90% mineral by volume thus becoming the most highly mineralized and hardest skeletal connective tissue. Rodent incisors erupt continuously to compensate for tooth wear caused by gnawing with newly differentiated ameloblasts appearing at the root apex to begin the incremental secretion of the enamel ECM as the tooth erupts. The complete process of enamel formation from initial matrix secretion to the eruption of mature enamel is therefore spatially laid out in a predictable sequence along the incisor, which makes the rodent incisor the ideal model to study all aspects of amelogenesis in a single tooth (27). The histological, SEM and CT data (Figs 4C, D and 6) indicate that female mice heterozygous for the p.Tyr64His amelogenin mutation secrete a biphasic enamel comprising an initial enamel layer ∼35 µm in thickness that is apparently normal. After this initial enamel layer is laid down, the ameloblasts become disorganized and any enamel elaborated after this point is abnormal, with reduced rod density, or is absent altogether. Assuming an enamel apposition rate of 6 µm per day (28) it can be estimated that the ameloblasts take ∼6 days to deposit the apparently normal initial 35 µm of enamel. During this time, the ameloblast monolayer remains intact and the enamel produced appears structurally normal. Presumably, during this period, the UPR is permissive for affected ameloblast survival and the integrity of the ameloblast monolayer is maintained. However, after this time, increasing pro-apoptotic signals in affected ameloblasts appear to tip the balance in favour of apoptosis resulting in a loss of those ameloblasts carrying the amelogenin mutation (∼50% of the ameloblasts are lost assuming random X-chromosome inactivation) and subsequent disruption of the ameloblast monolayer. This explains the apparent reduction in rod density in the enamel laid down on top of the initially secreted ∼35 µm layer (Fig. 6B) in affected animals since each rod is thought to be the product of a single ameloblast (26).

Having concluded that the p.Tyr64His phenotype was a result of induced ER stress and subsequent UPR-mediated ameloblast apoptosis, we hypothesized that 4-phenylbutyrate, which can act to relieve conformational disease pathology, would rescue the phenotype. 4-Phenylbutyrate has been investigated in clinical trials and *in vivo* and *in vitro* experimental studies as a potential therapeutic agent for the treatment of several diseases including cancer, spinal muscular atrophy, ischaemia, cystic fibrosis and Huntington's disease (13).

We found that COS-7 cells co-transfected with both p.Tyr64His amelogenin and ameloblastin underwent apoptosis but apoptosis was abolished with 4-phenylbutyrate treatment. Moreover, the known human amelogenin mutation Pro70Thr (15) also promoted apoptosis in COS-7 cells when co-expressed with ameloblastin. It has previously been suggested that human Pro70Thr amelogenin-linked AI is due to the mutation inhibiting enamel crystal growth in the enamel ECM based on *in vitro* data (29). Our COS-7 data suggest that ameloblast ER stress may also play a role in this specific example of human AI. The significance of the fact that mutated amelogenin is only apoptotic when co-expressed with ameloblastin is unclear.

Building on these cell transfection studies, we tested the efficacy of 4-phenylbutyrate in rescuing the p.Tyr64His phenotype *in vivo.* Following dietary administration of 4-phenylbutyrate, we observed a remarkable improvement in the phenotype of p.Tyr64His female mice, macroscopically, histologically and ultrastructurally. Importantly, the numbers of apoptotic cells were reduced to the level seen in wild-type mice. Restoration of the normal enamel architecture by 4-phenylbutyrate explains the observed improvement in enamel strength and the uniform undamaged appearance of the biting edge as the characteristic decussating structure dictates the physical properties of the tissue.

Previous reports indicate that 4-phenylbutyrate acts as a synthetic chaperone that directly interacts with the ER's secretory load to restore normal secretory function and a return to health (30,31). However, western blotting showed that 4-phenylbutyrate did not obviously improve the secretion of p.Tyr64His amelogenin into the ECM and the ECM remained deficient in full-length amelogenin compared with wild-type littermates. The results therefore suggest that 4-phenylbutyrate does not act as a chaperone in this mouse model of AI. Instead, 4-phenylbutyrate appears to inhibit ameloblast apoptosis, as indicated by its ability to return levels of affected ameloblasts immuno-positive for activated caspase 3 to wild-type levels.

In female heterozygous p.Tyr64His mice, 4-phenylbutyrate dramatically rescued the enamel phenotype even though only ∼50% of the ameloblasts would be expressing wild-type amelogenin and contributing to matrix deposition as the affected ameloblasts are still compromised in terms of amelogenin secretion. However, the inhibition of apoptosis in the affected ameloblasts by 4-phenylbutyrate preserves the structural integrity of the ameloblast monolayer allowing the cohort of wild-type ameloblasts to deposit a complete, structurally normal enamel layer in the presence of affected ameloblasts. In contrast, although 4-phenylbutyrate improved the ameloblast histology in male mice (hemizygous for p.Tyr64His amelogenin), the phenotype was not rescued presumably because all of the ameloblasts are affected and exhibit impaired amelogenin secretion.

How 4-phenylbutyrate promotes ameloblast survival over apoptosis in the presence of the mutation is unclear but 4-phenylbutyrate is a known histone deacetylase inhibitor (32) and can exert an impact on the transcription of multiple genes regulating apoptosis and also promote survival over apoptosis within certain cellular contexts (33–35). A scheme outlining the possible interactions of 4-phenylbutyrate with components of the UPR-induced apoptotic signalling pathway is presented in Supplementary Material, Figure S6.

In summary, our findings position this particular case of AI amongst the conformational diseases. As such, we present a new concept to explain the molecular pathogenesis of an inherited disease in a mouse model with direct relevance to the same condition in humans. We show that 4-phenylbutyrate can rescue the disease phenotype by inhibiting apoptosis in affected cells. Sodium-4-phenylbutyrate is FDA-approved and is well tolerated when administered at high doses as an ammonium scavenger in the treatment of human urea cycle disorders (13). Our observations therefore raise the possibility of treating at least a subset of human AI cases by administering sodium-4-phenylbutyrate from birth while the permanent dentition is developing. The murine AI model described here may also provide a useful tool to aid in our further understanding of ER stress in the future.

## MATERIALS AND METHODS

### Mice

The mutant mouse line M100888 was obtained from RIKEN GSC (http://www.gsc.riken.jp/Mouse/) and maintained on a DBA/2J genetic background. Control mice (*n* = 19) were fed a standard proprietary rodent diet (Special Diet Services, UK) while drug-treated mice (*n* = 22) were fed the same diet containing 7 g/kg sodium-4-phenybutyrate over a 10-week period. Mice were genotyped by PCR amplification of the genomic region containing *Amelx* exon 5 (forward primer sequence: 5′-ATGATAAGGCAGCCGGTATA-3′; reverse primer sequence: 5′-GTGATGAGGCTGAAGGGTGT-3′) followed by sequencing of the PCR product using dye primer chemistry (primer sequence: 5′-CACCTTCAAACACTAATGGG-3′). All experiments were performed in accordance with the Animals (Scientific Procedures) Act, UK, 1986. Unless otherwise stated, all experiments were performed using affected heterozygous female or affected hemizygous male *Amelx* mutant mice and their wild-type littermates.

### High-resolution immunofluorescence analysis

Following cervical dislocation, hemi-mandibles dissected from wild-type (*n* = 3) and affected male mice (*n* = 3) were fixed in 4% paraformaldehyde in 0.1 m cacodylate buffer (pH 7.4) overnight at 4°C, rinsed in phosphate-buffered saline (PBS) and demineralized for 3 weeks in 0.5 m EDTA, pH 7.0. Following demineralization, samples were re-fixed with 4% paraformaldehyde in 0.1 m cacodylate buffer (pH 7.4) overnight. Next morning, samples were dehydrated through a graded ethanol series and embedded as transverse segments, corresponding to the secretory and maturation regions of the tooth, in acrylic resin (LR White) and polymerized at 40°C for 24 h. Semi-thin sections were prepared using a glass knife and mounted on Superfrost Plus glass slides (Thermo Shandon).

Dual immunofluorescence was performed for amelogenin (FL191, Santa Cruz Biotechnology) and ameloblastin (C17, Santa Cruz Biotechnology). Primary antibodies were detected using Alexa Fluor 488 (amelogenin) and Alexa Fluor 555 (ameloblastin) conjugated secondary antibodies (Invitrogen) and mounted in Mowiol 4–88 mounting medium containing 2.5% DABCO and 0.1% DAPI. Immunolabelled sections were examined on a Delta Vision RT (Applied Precision) restoration microscope. Images were collected using a Coolsnap HQ (Photometrics) camera with a Z optical spacing of 0.2 μm. Raw images were then deconvolved using Softworx software and displayed as maximum intensity projections.

### Transmission electron microscopy

Two-month-old mouse mandibles (four wild-type male and four affected male mice) were dissected following cervical dislocation and fixed in 2% paraformaldehyde/2% glutaraldehyde prepared in 0.1 m cacodylate buffer containing 0.15 m sucrose and 2 mm calcium chloride (pH 7.3) at 4°C overnight. Hemi-mandibles were demineralized as above and dissected axially into posterior, medial and anterior portions. Following dissection, the samples were washed with cacodylate buffer, post-fixed in 1% osmium tetroxide, dehydrated through a graded ethanol series, cleared in propylene oxide and embedded in Epoxy resin (100 resin, Agar Scientific Ltd.). Ultrathin sections were contrasted with uranyl acetate and lead citrate, and examined on a Philips model 400 transmission electron microscope.

### Immunofluorescence analysis of the enamel organ

Mandibles from 3-month-old affected male mice and wild-type mice (*n* = 4 in each category) were dissected following cervical dislocation and fixed in 4% paraformaldehyde dissolved in PBS (pH 7.4) for 24 h. Following fixation, the mandibles were demineralized as above, dehydrated through a graded ethanol series, cleared in chloroform, embedded as hemi-mandibles in paraffin wax and sectioned onto Superfrost Plus glass slides (Thermo Shandon). Immunofluorescence was performed using primary antibodies to amelogenin (FL191; Santa Cruz Biotechnology), calnexin (C4731; Sigma-Aldrich), protein disulphide isomerase (P7496; Sigma-Aldrich), ER-Golgi intermediate compartment protein 53 kDa (ERGIC 53, sc-66880; Santa Cruz Biotechnology), *cis*-Golgi matrix protein 130 kDa (GM 130, sc-55590; Santa Cruz Biotechnology), Golgin 245 (sc-102565; Santa Cruz Biotechnology) and activated caspase 3 (AF835; R & D Systems). The rabbit anti-β-COP antibody, EAGE, was raised in the laboratory of Professor Thomas Kreis (deceased) and provided by Martin Lowe (University of Manchester). Primary antibodies were detected using Alexa Fluor 488- or Alexa Fluor 555-conjugated secondary antibodies (Invitrogen) and mounted in Mowiol 4–88 mounting medium containing 2.5% DABCO and 0.1% DAPI. Images were collected using deconvolution microscopy as described above.

### *In situ* hybridization and quantitative PCR analysis of ER stress markers

A 350 bp fragment of BiP cDNA (IMAGE clone ID6334883) was cloned into pBluescript SK (Stratagene) and the resulting construct used to synthesize digoxygenin-labelled sense and antisense riboprobes using digoxygenin-conjugated dUTP (Roche) and T7 and T3 RNA polymerase (Promega), respectively. *In situ* hybridization was performed as described previously (36,37); however, riboprobe binding was detected immunohistochemically using an alkaline phosphatase-conjugated, anti-digoxygenin antibody (Roche) followed by histochemical demonstration of alkaline phosphatase activity using BM purple (Roche) as the chromogenic substrate. Endogenous alkaline phosphatase activity was inhibited using levamisole. Paraffin wax-processed sections (6 µm) of three hemi-mandibles from affected female, affected male and wild-type mice were examined.

For quantitative PCR analysis total RNA was extracted using the RNeasy kit (Qiagen, Crawley, UK) from micro-dissected secretory-stage enamel organs (4 wild-type, 4 homozygous female mice), quantified using a NanoDrop 2000 spectrophotometer (ThermoShandon), and reverse transcribed to complementary DNA. Quantitative reverse transcriptase–PCR was performed according to the manufacturer's instructions on a StepOne Plus machine using SYBR Green master mix (Life Technologies, Paisley, UK) and analysed using the ΔΔ-Ct method, normalized to β-actin. Results were analysed using a one-tailed Mann–Whitney *U*-test. The following primer pairs were used for the analysis: *hspa5* 5′-ATCTTTGGTTGCTTGTCGCT-3′, 5′-ATGAAGGAGACTGCTGAGGC-3′; *Xbp1* 5′-CCGTGAGTTTTCTCCCGTAA-3′, 5′-AGAAAGAAAGCCCGGATGAG-3′; *Hsp90b1* 5′-TTGTGTCCAATTCAAGGTAATCA-3′, 5′-TTGCTGACCCAAGAGGAAAC-3′; *Atf4* 5′-TTGTCCGTTACAGCAACACTG-3′, 5′-GCAGCAGCACCAGGCTCT-3′; *Ddit3* 5′-GACCAGGTTCTGCTTTCAGG-3′, 5′-CAGCGACAGAGCCAGAATAA-3′.

### Western blotting

Following cervical dislocation, mandibular incisor teeth were dissected free of the mandibular bone and the enamel organ gently wiped off the enamel surface with a moist paper tissue. Enamel corresponding to the secretory stage was dissected from the underlying dentine and immediately frozen in liquid nitrogen prior to storage at −80°C. Enamel samples were extracted directly into 25 µl of SDS–PAGE sample loading buffer (38) containing a cocktail of enzyme inhibitors (Roche). Samples were heated at 90°C for 2 min and loaded onto 12% SDS–PAGE gels (Bio-Rad mini protean III) and electrophoresed at 200 V. Following SDS–PAGE gels were electro-blotted onto nitrocellulose at 80 V for 1 h. Blots were blocked by overnight incubation at 4°C in 5% milk powder in TBS. After blocking, blots were incubated with primary antibody raised in rabbit against full-length human recombinant amelogenin (Santa Cruz SC-32892) diluted 1:1000 in TBS + 0.05% Tween 20 (TBST) for 1 h at room temperature. Excess antibody was removed by washing with TBST (3 × 5 min). The blot was incubated with anti-rabbit IgG peroxidase conjugate (Sigma A0545) diluted 1:2000 for 1 h at room temperature. Excess antibody was removed by washing with TBST (5 × 5 min) and immuno-reactivity revealed by staining with metal-enhanced DAB (Sigma D0426) used in accordance with the manufacturer's instructions.

### High-resolution X-ray CT

Following dissection of secretory stage enamel, incisor teeth were glued to glass microscope slides and mounted in polypropylene tubes for CT scanning. Scans were obtained using a high- resolution X-ray CT system (Phoenix Nanotom 160 NF GE, Germany) operated at a tube voltage of 80 kV, current 100 µA and irradiation time of 1000 ms. Images were recorded as the sample was rotated through 360° in incremental steps of 0.25° generating a total of 1440 images. Resolution was 7 µm per voxel. A transverse section through a mature incisor from a wild-type mouse was used as an internal calibration standard. The mineral density of the standard had been previously determined by transverse microradiography.

Resulting scans were analysed and processed using ImageJ software.

### Scanning electron microscopy

Incisors previously subjected to X-ray CT scanning were ground with fine carborundum paper to obtain transverse sections through the incisor ∼2 mm from the incisal tip. Any smear layer was removed by etching the ground surface in 30% phosphoric acid for 20 s followed by thorough rinsing in excess distilled water. Teeth were dried overnight under vacuum and sputter coated with gold. Specimens were observed using a Hitachi S-3400N scanning electron microscope operated at an accelerating voltage of 20 kV and an emission current of 82 µA.

### Transfection experiments

COS-7 cells were cultured on coverslips in DMEM containing 10% fetal bovine serum at 37°C and 5% CO_2_. Once cells had reached 80% confluence, single transfections and co-transfections were established using the wild-type amelogenin, p.Tyr64His mutant amelogenin and wild-type ameloblastin expression constructs described previously (9). An expression construct containing the Pro70Thr mutation which causes AI in humans was made by engineering the mutation into the wild-type expression construct as described previously (9). Each transfection experiment was performed in triplicate. Transfection was achieved using Lipofectamine 2000 according to the manufacturer's instructions (Invitrogen) and cultures were incubated as above in the presence or absence of 0.5 mm sodium-4-phenylbutyrate for 18 h. Transfections omitting the DNA constructs were established in parallel as negative controls. Subsequently, cells were fixed in ice cold 4% paraformaldehyde and made permeable using 0.1% Triton X-100 in PBS. Cell death was detected by TUNEL assay using the ‘*In Situ* Cell Death’ Kit (Roche) according to the manufacturer's instructions. Three contiguous images of each sample were collected using the ×10 objective lens of a DMRB microscope (Leica) and Spot™ digital camera and associated software (RTKE/SE Diagnostic Instruments Inc.). The percentage of TUNEL-positive cells in each image was determined using the ‘Analyse Particles’ feature of ImageJ image analysis software (Rasband, W.S., ImageJ, US National Institutes of Health, Bethesda, MD, USA, http://imagej.nih.gov/ij/, 1997–2012). Results were analysed using the Kruskal–Wallis test followed by a Dunn's multiple comparison test using GraphPad Prism^®^ v5.0 software (GraphPad, San Diego, CA, USA).

### Morphometric procedures

Measurements of the secretory stage enamel organ were carried out on paraffin wax-processed, haematoxylin and eosin-stained, longitudinal sections (6 µm), cut through the midline of the mandible. The distance from where the enamel matrix could first be detected, at the apical region of the tooth, and the point, distally, where the ameloblasts first became disorganized was measured using ImageJ image analysis software (see above). In wild-type mice, the most distal measurement was the transition stage immediately prior to the commencement of the maturation stage of amelogenesis. In each section, the greatest thickness of secreted enamel matrix was also measured over this distance. Hemi-mandibles of seven untreated affected female mice, 4-phenylbutyrate-treated affected female mice, six untreated affected male mice and eight 4-phenylbutyrate-treated affected male mice were analysed in this way. Results were analysed using the Mann–Whitney *U*-test using GraphPad Prism software (see above).

Measurements of intracellular vesicle areas were performed on paraffin wax-processed, longitudinal sections (6 µm). Ameloblastin was immunolabelled in these sections to demonstrate the vesicles and three images of each specimen were collected using deconvolution microscopy as described above. The ‘Analyse Particles’ feature of Image J image analysis software (see above) was used to obtain the areas of individual intracellular vesicles. Four hemi-mandibles of untreated and 4-phenylbutyrate-treated affected female mice and affected male mice were analysed in this way. Results were analysed by the Kruskal–Wallis test followed by a Dunn's multiple comparison test using GraphPad Prism software (see above).

### Statistical analyses

Statistical differences between the experimental groups in the transfection analyses and the vesicle measurements were analysed using the non-parametric Kruskal–Wallis one-way analysis of variance followed by Dunn's multiple comparisons post-test. Qualitative assessment of the appearance of affected female mouse enamel from images taken at the beginning and end of the 4-phenylbutyrate-treatment experiment by seven-independent assessors was analysed using Fisher's exact test. Measurements of the secretory stage enamel organ and enamel matrix thickness were compared using the two-tailed Mann–Whitney *U*-test. Comparison of gene expression by qPCR was analysed using a one-tailed Mann–Whitney *U*-test. In all statistical analyses, *α* = 0.05.

## SUPPLEMENTARY MATERIAL

Supplementary Material is available at *HMG* online.

## FUNDING

The authors wish to acknowledge the support of Wellcome Trust programme grant 075945 and the Wellcome Trust Institutional Strategic Support Fund (097820). J.K. is supported by the Leeds NIHR Musculoskeletal Biomedical Research Unit and WELMEC: A Centre of Excellence in Medical Engineering, funded by the Wellcome Trust and EPSRC, under Wellcome Trust grant number 088908. Funding to pay the Open Access publication charges for this article was provided by the Wellcome Trust UK.

## Supplementary Material

Supplementary Data
