# Supplementary material for: Endoplasmic reticulum stress in amelogenesis imperfecta and phenotypic rescue using 4-phenylbutyrate
Source: Hum Mol Genet. 2013 Dec 20;23(9):2468–80. doi: 10.1093/hmg/ddt642 (PMC3976337; doi:10.1093/hmg/ddt642)
Supplement: Supplementary Data [file supp_23_9_2468__index.html]

Endoplasmic reticulum stress in amelogenesis imperfecta and phenotypic rescue using 4-phenylbutyrate — Endoplasmic reticulum stress in amelogenesis imperfecta and phenotypic rescue using 4-phenylbutyrate — Supplementary Data 

# Endoplasmic reticulum stress in *amelogenesis imperfecta* and phenotypic rescue using 4-phenylbutyrate

## Supplementary Data

Supplementary Data

**Files in this Data Supplement:**

- Supplementary Data - Pdf file
- Supplementary Video - avi file
