# Supplementary material for: Endoplasmic reticulum stress in amelogenesis imperfecta and phenotypic rescue using 4-phenylbutyrate
Source: Hum Mol Genet. 2013 Dec 20;23(9):2468–80. doi: 10.1093/hmg/ddt642 (PMC3976337; doi:10.1093/hmg/ddt642)
Supplement: Supplementary Data [file supp_ddt642_ddt642supp.pdf]

Supplementary Material

Figure S1. **Clinical appearance of a case of autosomal recessive *amelogenesis imperfecta.*** The enamel is discoloured and hypoplastic. Where enamel is absent, exposure of the underlying dentinal tubules leads to dentine sensitivity and pain in response to heat, cold and chemical stimuli.

Figure S2. Characterization of secretory pathway molecules observed in wild-type male mice. (**A-F**) Dual immuno-staining for amelogenin (red fluorescence) or (**G-I**) ameloblastin (red fluorescence) and components of the secretory pathway (green fluorescence) in wild-type male mice (n = 4). (A) The ER membrane protein calnexin appears throughout the ameloblast cytoplasm, as does (B) the ER luminal molecule protein disulphide isomerase (PDI) .(C) Immuno-reactivity for the ER/Golgi intermediate compartment molecule Ergic 53 shows a reticular pattern in the ameloblast cytoplasm and frequently appears to co-localize with amelogenin (yellow fluorescence, arrows). (D) β-COP, a component of COP1 vesicles found at the *cis*-Golgi and ER/Golgi intermediate compartment shows a similar distribution to Ergic 53 but does not appear to co-localize with amelogenin. (E) The protein GM130 appears to occupy a central position within the ameloblast cytoplasm consistent with it being a component of the *cis*-Golgi and frequently co-localizes with amelogenin. (F) Golgin 245, a *trans*-Golgi protein, shows a reticular pattern of immuno-reactivity. (G) Dual immunofluorescence for ameloblastin and the ER membrane protein calnexin, shows widespread calnexin immuno-reactivity throughout the ameloblast cytoplasm. (H) Analysis using antibodies directed against ameloblastin and the ER luminal molecule protein disulphide isomerase (PDI) shows a similar distribution to that seen in (B). (**I**) In contrast, dual immunofluorescence with ameloblastin and Ergic 53, a protein found in the ER/Golgi intermediate compartment, demonstrates distinct

co-localization within the ameloblasts (arrows) and particularly in the Tomes' processes (red arrow). Nuclei are stained with DAPI (blue). Scale bars: (A-F) 5 µm (G-H) 10 µm .

Figure S3. 4-Phenylbutyrate abrogates the apoptosis induced by expression of p.Tyr64His and p.Pro70Thr recombinant amelogenins. (**A**) Cells transfected with constructs expressing wild-type amelogenin, amelogenin carrying the p.Tyr64His mutation, wild-type ameloblastin, or wild-type amelogenin together with wild-type ameloblastin exhibited low levels of cell death that were not significantly affected by incubation with 4-phenylbutyrate. In contrast, COS-7 cells simultaneously expressing mutant amelogenin and wild-type ameloblastin exhibited a statistically significant increase in the mean percentage of TUNEL-positive nuclei ($p < 0.0001$) that was significantly abrogated by 4-phenylbutyrate ($p < 0.0001$). (**B**) Effect of 4-phenylbutyrate treatment on COS-7 cells transfected with a construct expressing amelogenin containing the mutation p.Pro70Thr. Cells transfected with the p.Pro70Thr amelogenin construct alone show low levels of apoptosis which is increased in cells simultaneously expressing mutant amelogenin and wild-type ameloblastin. Treatment of COS-7 cells with the p.Pro70Thr amelogenin construct produced levels of apoptosis which did not differ significantly from those seen in cells treated with the wild-type amelogenin construct while treatment of COS-7 cells expressing the p.Pro70Thr amelogenin construct with 0.5 mM PB resulted in a statistically significant reduction in the incidence of cell death ($p = 0.0097$). Simultaneous expression of p.Pro70Thr amelogenin and wild-type ameloblastin caused a significant increase in apoptosis ($p = 0.0039$) compared to COS-7 cells expressing wild-type amelogenin. Similarly, PB-treatment of COS-7 cells simultaneously expressing mutant amelogenin and wild-type ameloblastin resulted in

a highly significant reduction in apoptosis (p = 0.0005). Error bars in A and B represent the standard deviation of the mean for each dataset. Each transfection experiment was performed three times. Abbreviations: Ambn, wild-type ameloblastin expression construct; Wt, wild-type amelogenin expression construct; Tyr64His, *Amelx* p.Tyr64His expression construct; Pro70Thr, p.Pro70Thr amelogenin expression construct; PB, 4-phenylbutyrate.

Figure S4. Effect of 4-phenylbutyrate treatment on the secretory zone morphology in affected mice. Treatment of affected female and male mice with 4-phenylbutyrate increased the length of the intact secretory zone (brackets) and shifted the region of maximum cell death towards the incisal tip (asterisk). Scale bars 100 µm.

Figure S5. Effect of 4-phenylbutyrate treatment in male mice. *Amelx* is present on the X-chromosome and as a result males are hemizygous for the p.Tyr64His amelogenin mutation, exhibiting a more severe phenotype than heterozygous females since all ameloblasts are affected. (**A**) Morphometric analysis of the secretory stage enamel organ of affected male mice. The distance over which secretory stage ameloblasts remained organised and the maximum enamel thickness achieved over this distance in untreated and PB-treated affected male mice were compared. In untreated mice, the distance over which the secretory stage enamel organ remained intact (left graph) was significantly less than the equivalent distance in PB-treated mice (p = 0.0047). Similarly, in untreated mice (n = 7), the enamel thickness deposited by the organised secretory stage enamel organ (right graph) was significantly less than the equivalent thickness in PB-treated mice ((n = 7) (p = 0.0093)). Error bars represent the standard deviation of the mean for all datasets. (**B-**

C) Activated caspase 3 immuno-staining was used to identify secretory stage ameloblasts undergoing apoptosis in untreated and PB-treated affected male mice. In untreated mice (B), numerous apoptotic cells were observed (arrows) while in the PB-treated affected male mice (C), fewer apoptotic cells were detected (arrows). Three mice were examined in each group. Scale bars: 100 µm. (**D**) SEM and CT scans (calibrated for mineral density) of wild-type enamel exhibiting the classic rodent decussating prism pattern. (**E**) Affected male mouse enamel, where present, is biphasic with a thin inner layer showing evidence of prism decussation and a disorganized outer layer. (**F**) Treatment with PB fails to rescue the affected male mouse phenotype. Three mice were examined in each group.

Figure S6. Diagram showing possible impact of PB on UPR/apoptosis cell signalling pathways. Western blotting of secreted matrix proteins (Fig 5B) suggests that PB does not relieve secretory impairment. Instead, PB might rescue the phenotype by inhibiting apoptosis and promoting cell survival by modulating UPR and apoptotic signalling pathways: Activation of ER stress sensor IRE1 (1) leads to JNK mediated phosphorylation (inactivation) of Bcl-2/Bcl-xl which are otherwise anti-apoptotic due to their ability to repress Bax/Bak modulated mitochondrial membrane permeabilization and JNK phosphorylation of Bid/Bim (2) which then instigates apoptosis by promoting Bax/Bak modulated mitochondrial membrane permeabilization (3). PB can prevent JNK activation thus favouring survival. Activation of stress sensor PERK (4) causes phosphorylation (activation) of eIF2 which induces CHOP expression via transcription factor ATF4 (5). CHOP favours apoptosis by down-regulating expression of anti-apoptotic Bcl-2/Bcl-xl and up-regulating the expression of pro-apoptotic Bid/Bim (6). PB can negatively modulate phosphorylated eIF2 and CHOP thus favouring survival. PB promotes the release of

the transcription factor NF–kB from its inhibitory modulator IkB (7). NF–kB is activated following acetylation by histone acetyltransferases (HAT). PB inhibits histone deacetylase (HDAC) activity (8) thus enhancing NF-kB activation of anti-apoptotic factors Bcl-2/Bcl-xl. Acetylated NF-kB also positively modulates a number of target molecules (e.g. C-FLIP) that inhibit the apoptotic caspase cascade induced by the mitochondrial release of cytochrome- c (10). Finally, PB up-regulates expression of HSP 70 which can inhibit the apoptotic caspase cascade (11) and block the pro-apoptotic activity of Bid/Bim interactions with Bax/Bak (12). Note that PB can be both anti- and pro-apoptotic (as can NF-kB) depending on cell context and the scheme outlined here is intended to introduce possible mechanisms by which PB may inhibit apoptosis in secretory stage ameloblasts expressing p.Tyr64His amelogenin (compiled with reference to (1-7)).

1. Ryu, H., Smith, K., Camelo, S.I., Carreras, I., Lee, J., Iglesias, A.H., Dangond, F., Cormier, K.A., Cudkowicz, M.E., Brown, R.H., Jr. *et al.* (2005) Sodium phenylbutyrate prolongs survival and regulates expression of anti-apoptotic genes in transgenic amyotrophic lateral sclerosis mice. *J. Neurochem.*, **93**, 1087-1098.

2. Vilatoba, M., Eckstein, C., Bilbao, G., Smyth, C.A., Jenkins, S., Thompson, J.A., Eckhoff, D.E. and Contreras, J.L. (2005) Sodium 4-phenylbutyrate protects against liver ischemia reperfusion injury by inhibition of endoplasmic reticulum-stress mediated apoptosis. *Surgery*, **138**, 342-351.

3. Creagh, E.M., Carmody, R.J. and Cotter, T.G. (2000) Heat shock protein 70 inhibits caspase-dependent and -independent apoptosis in Jurkat T cells. *Exp. Cell Res.*, **257**, 58-66.

4. Gong, B., Zhang, L.Y., Lam, D.S., Pang, C.P. and Yam, G.H. (2010) Sodium 4-phenylbutyrate ameliorates the effects of cataract-causing mutant gammaD-crystallin in cultured cells. *Mol. Vis.*, **16**, 997-1003.

5. Kim, D.S., Li, B., Rhew, K.Y., Oh, H.W., Lim, H.D., Lee, W., Chae, H.J. and Kim, H.R. (2012) The regulatory mechanism of 4-phenylbutyric acid against ER stress-induced autophagy in human gingival fibroblasts. *Arch. Pharm. Res.*, **35**, 1269-1278.

6. Li, C.Y., Lee, J.S., Ko, Y.G., Kim, J.I. and Seo, J.S. (2000) Heat shock protein 70 inhibits apoptosis downstream of cytochrome c release and upstream of caspase-3 activation. *J. Biol. Chem.*, **275**, 25665-25671.

7. Ren, D., Tu, H.C., Kim, H., Wang, G.X., Bean, G.R., Takeuchi, O., Jeffers, J.R., Zambetti, G.P., Hsieh, J.J. and Cheng, E.H. (2010) BID, BIM, and PUMA are essential for activation of the BAX- and BAK-dependent cell death program. *Science*, **330**, 1390-1393.

Video S1. Video cycling through transverse CT image stacks of affected incisor teeth of affected female ($Amelx^{X/Tyr64His}$) mice compared to those formed under the influence of 4-phenylbutyrate

Figure S1

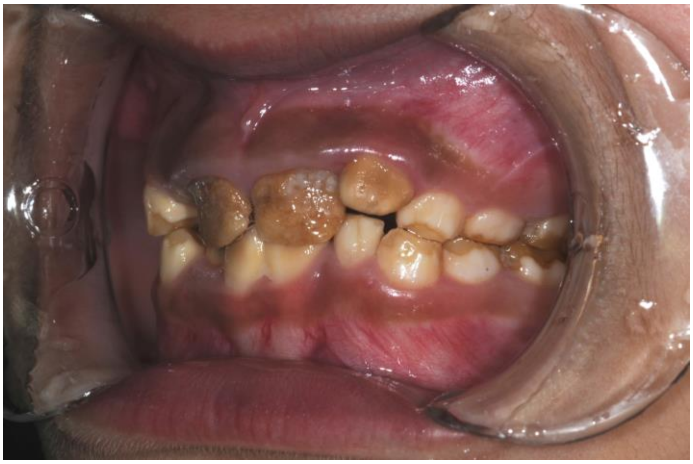

Figure S2

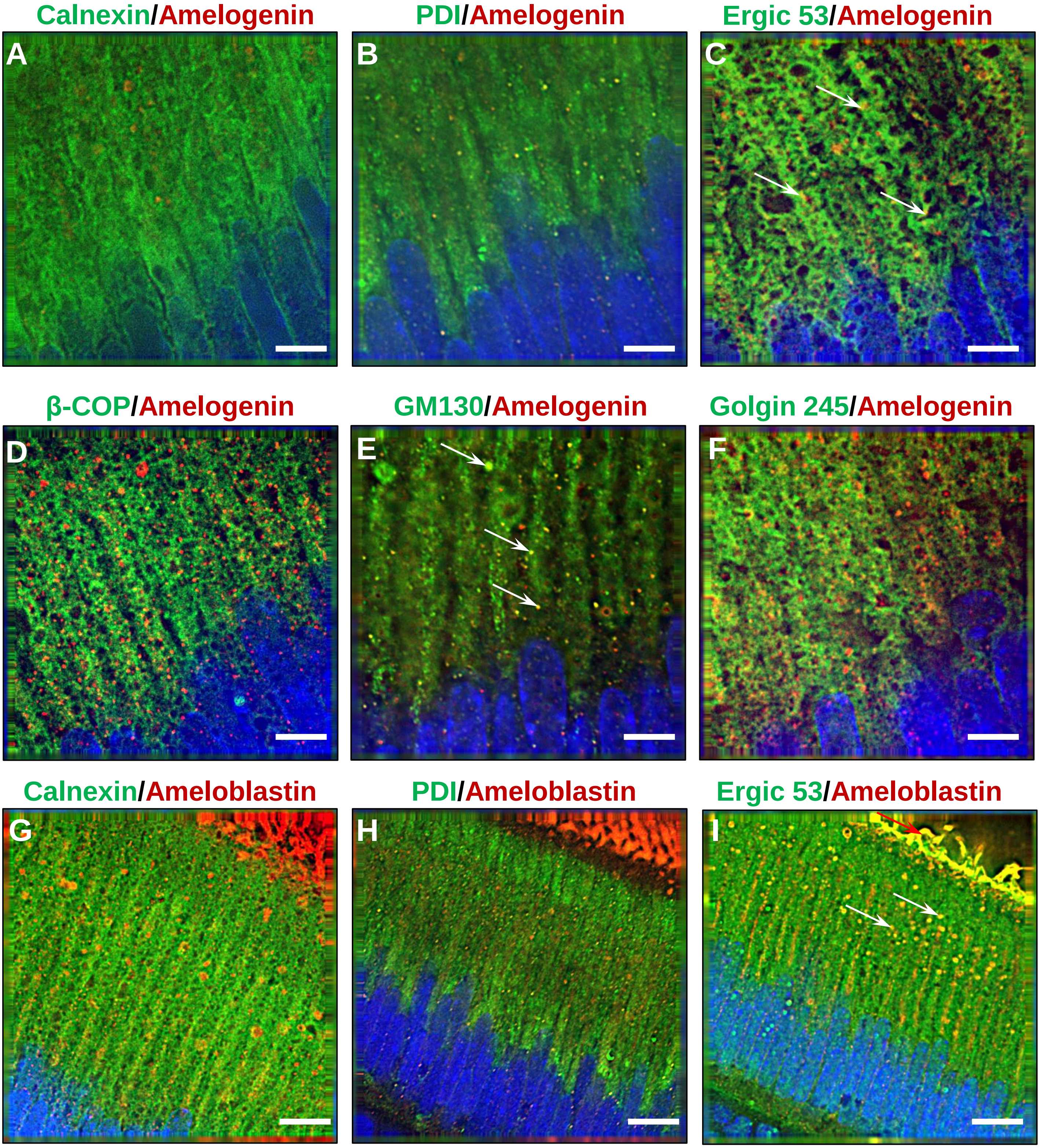

Calnexin/Amelogenin
A
PDI/Amelogenin
B
Ergic 53/Amelogenin
C
β-COP/Amelogenin
D
GM130/Amelogenin
E
Golgin 245/Amelogenin
F
Calnexin/Ameloblastin
G
PDI/Ameloblastin
H
Ergic 53/Ameloblastin
I

Figure S3

A

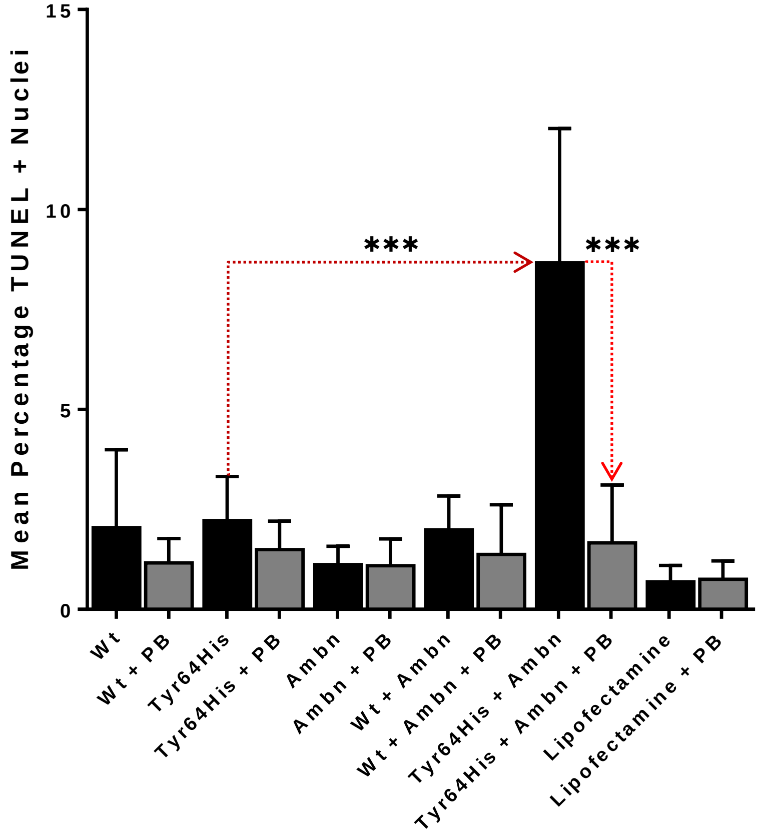
Mean Percentage TUNEL + Nuclei
15
10
5
0
***
***
Wt
Wt + PB
Tyr64His
Tyr64His + PB
Ambn
Ambn + PB
Wt + Ambn
Wt + Ambn + PB
Tyr64His + Ambn
Tyr64His + Ambn + PB
Lipofectamine
Lipofectamine + PB

B

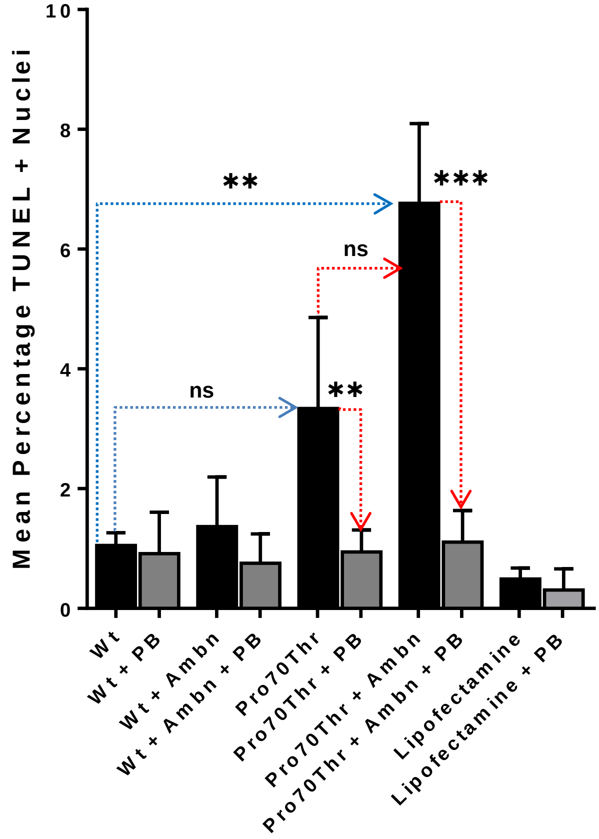
Mean Percentage TUNEL + Nuclei
10
8
6
4
2
0
**
***
ns
ns
**
Wt
Wt + PB
Wt + Ambn
Wt + Ambn + PB
Pro70Thr
Pro70Thr + PB
Pro70Thr + Ambn
Pro70Thr + Ambn + PB
Lipofectamine
Lipofectamine + PB

Figure S4

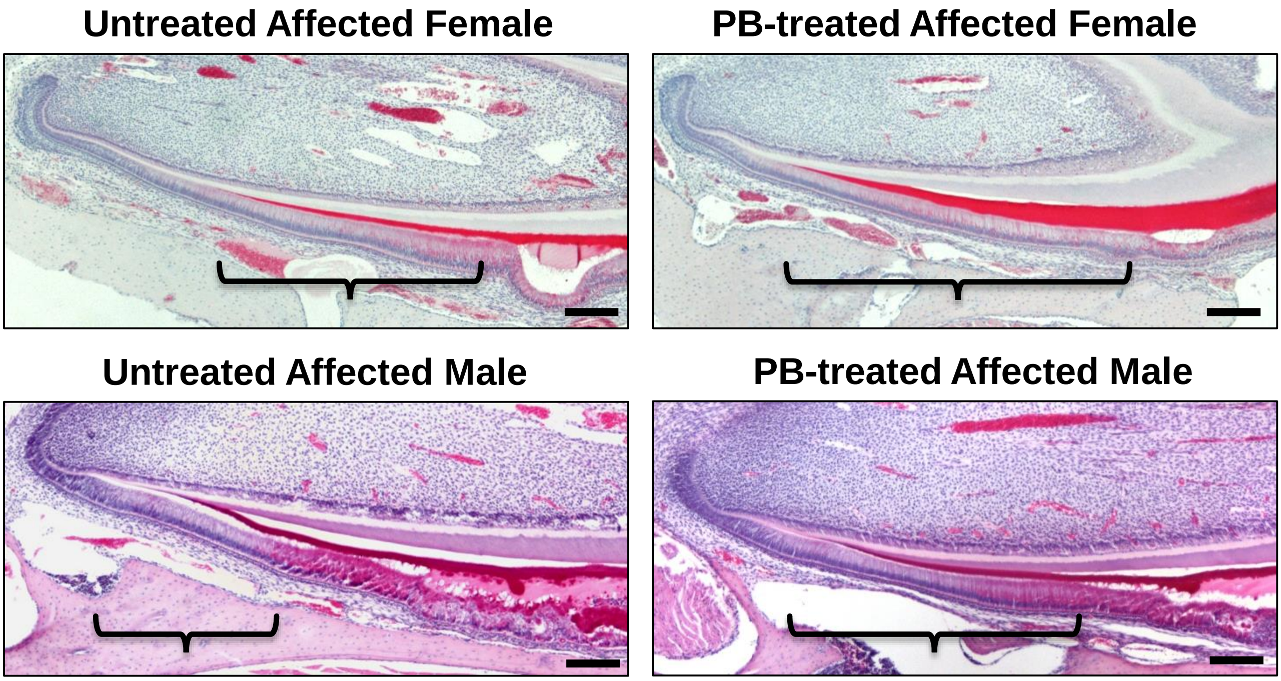
Untreated Affected Female
PB-treated Affected Female
Untreated Affected Male
PB-treated Affected Male

Figure S5

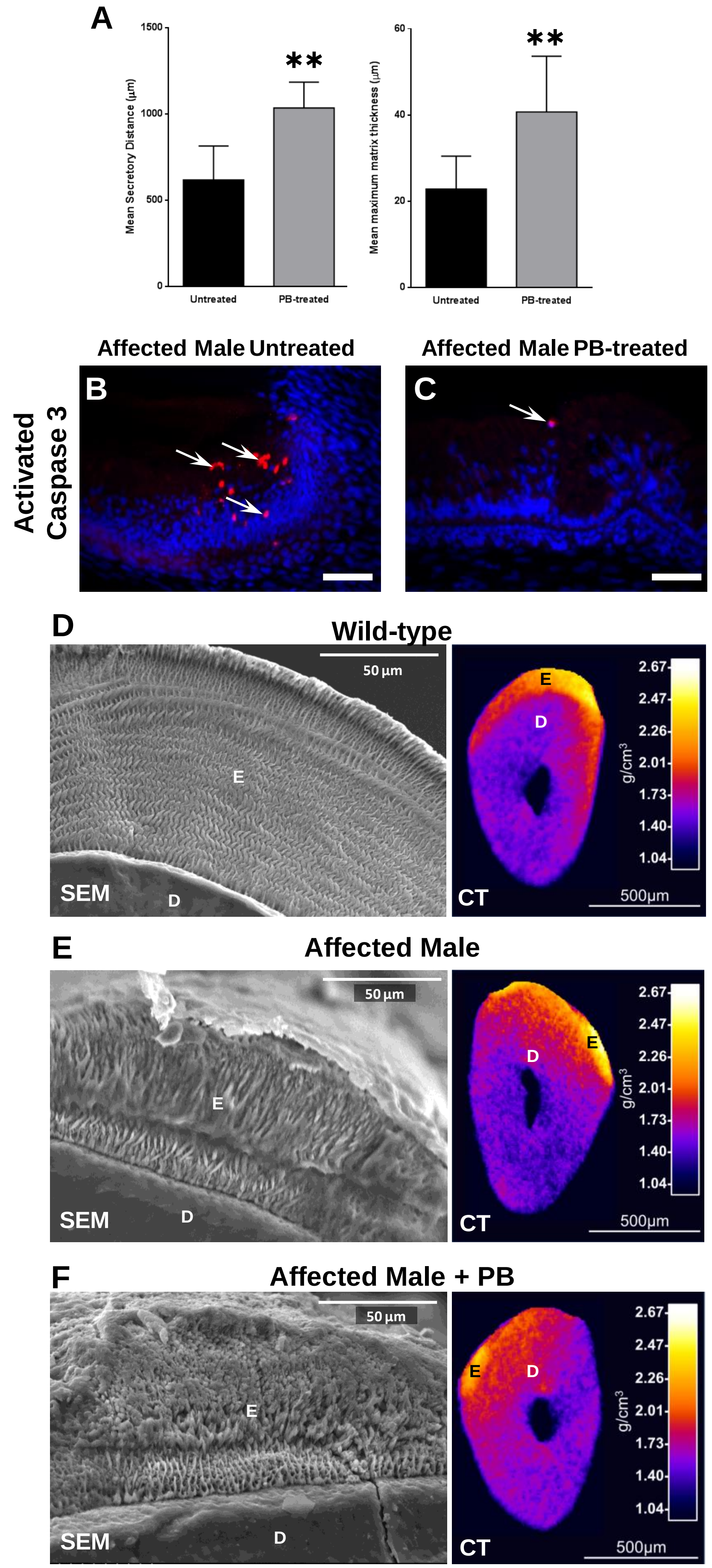

A
Mean Secretory Distance (µm)
1500
1000
500
0
**
Untreated
PB-treated
Mean maximum matrix thickness (µm)
60
40
20
0
**
Untreated
PB-treated
Affected Male Untreated
Affected Male PB-treated
Activated Caspase 3
B
C
D
Wild-type
50 µm
E
SEM
D
E
D
CT
2.67
2.47
2.26
2.01
1.73
1.40
1.04
g/cm3
500µm
E
Affected Male
50 µm
E
SEM
D
D
E
CT
2.67
2.47
2.26
2.01
1.73
1.40
1.04
g/cm3
500µm
F
Affected Male + PB
50 µm
E
SEM
D
E
D
CT
2.67
2.47
2.26
2.01
1.73
1.40
1.04
g/cm3
500µm

Figure S6

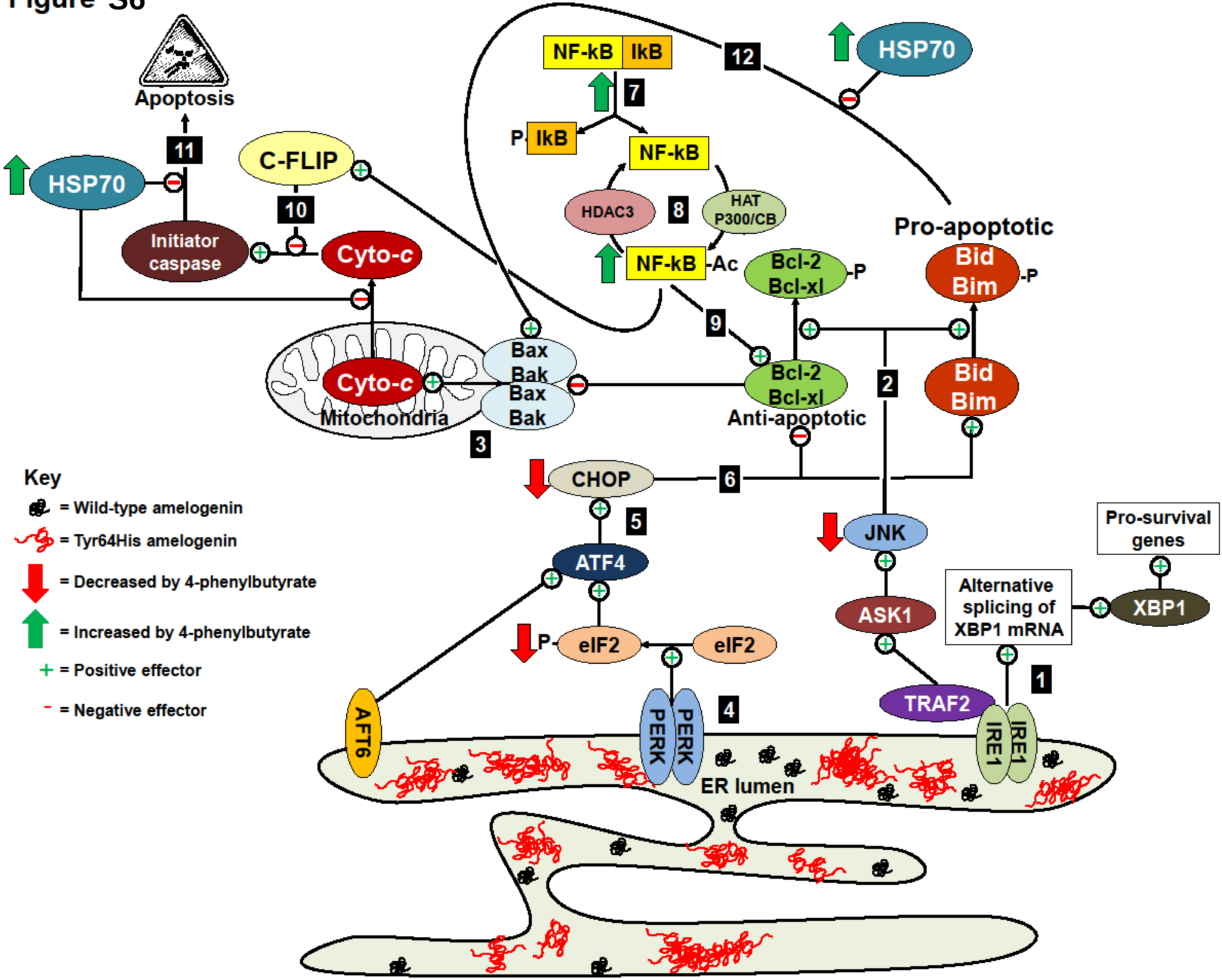

Apoptosis
11
HSP70
C-FLIP
10
Initiator caspase
Cyto-c
Cyto-c
Mitochondria
Bax Bak
Bax Bak
3
NF-kB
IkB
12
HSP70
7
P-IkB
NF-kB
HDAC3
8
HAT P300/CB
NF-kB-Ac
Bcl-2 Bcl-xl -P
Pro-apoptotic
Bid Bim -P
9
Bcl-2 Bcl-xl
Anti-apoptotic
2
Bid Bim
CHOP
6
5
ATF4
JNK
Pro-survival genes
ASK1
Alternative splicing of XBP1 mRNA
XBP1
P-eIF2
eIF2
1
TRAF2
4
AFT6
PERK
PERK
IRE1
IRE1
ER lumen
Key
= Wild-type amelogenin
= Tyr64His amelogenin
= Decreased by 4-phenylbutyrate
= Increased by 4-phenylbutyrate
+ = Positive effector
- = Negative effector
